# Substrate induced nanoscale resistance variation in epitaxial graphene

**DOI:** 10.1038/s41467-019-14192-0

**Published:** 2020-01-28

**Authors:** Anna Sinterhauf, Georg A. Traeger, Davood Momeni Pakdehi, Philip Schädlich, Philip Willke, Florian Speck, Thomas Seyller, Christoph Tegenkamp, Klaus Pierz, Hans Werner Schumacher, Martin Wenderoth

**Affiliations:** 1 IV. Physikalisches Institut, Georg-August-Universität Göttingen, Friedrich-Hund-Platz 1, 37077 Göttingen, Germany; 2 Physikalisch-Technische Bundesanstalt, Bundesallee 100, 38116 Braunschweig, Germany; 3 Institut für Physik, Technische Universität Chemnitz, Reichenhainer Straße 70, 09126 Chemnitz, Germany; 4 Zentrum für Materialien, Architekturen und Integration von Nanomembranen (MAIN), Technische Universität Chemnitz, Rosenbergstraße 6, 09126 Chemnitz, Germany; 5 Center for Quantum Nanoscience, Institute for Basic Science (IBS), Seoul, 03760 Republic of Korea; 6 Ewha Womans University, Seoul, 03760 Republic of Korea

**Keywords:** Electronic properties and devices, Surfaces, interfaces and thin films

## Abstract

Graphene, the first true two-dimensional material, still reveals the most remarkable transport properties among the growing class of two-dimensional materials. Although many studies have investigated fundamental scattering processes, the surprisingly large variation in the experimentally determined resistances is still an open issue. Here, we quantitatively investigate local transport properties of graphene prepared by polymer assisted sublimation growth using scanning tunneling potentiometry. These samples exhibit a spatially homogeneous current density, which allows to analyze variations in the local electrochemical potential with high precision. We utilize this possibility by examining the local sheet resistance finding a significant variation of up to 270% at low temperatures. We identify a correlation of the sheet resistance with the stacking sequence of the 6H silicon carbide substrate and with the distance between the graphene and the substrate. Our results experimentally quantify the impact of the graphene-substrate interaction on the local transport properties of graphene.

## Abstract (web summary)

Measurement of charge transport in epitaxial graphene is challenging. Here, the authors quantitatively investigate local transport properties of graphene prepared by polymer assisted sublimation growth using scanning tunneling potentiometry and report local sheet resistances with a variation of up to 270% at low temperatures.

## Introduction

Charge transport in epitaxial graphene has been subject of theoretical and experimental investigation since its first electronic characterization^1^. The high quality and its 2D nature make epitaxial graphene the perfect system to study fundamental transport properties on the nanometer scale. Consequently, in a series of experiments—based on scanning tunneling potentiometry (STP)^2^ or four-point-probe microscopy^3^—several groups have focused on local properties like the sheet resistance and the impact of scattering centers like single substrate steps^4,5^ or the transition from monolayer to bilayer graphene on transport^6,7^. From these results, it is qualitatively well understood that the transport properties of epitaxial graphene are not homogeneous on the nanometer scale. Substrate steps or monolayer-bilayer junctions act as local scattering centers. In addition, for epitaxial graphene on SiC(0001) it is well known that interaction with the substrate drastically affects graphene’s transport properties. In order to reduce this inherent proximity effect, i.e., to improve the transport properties of the graphene sheet, different strategies were pursued such as the refinement of the growth process^8–10^, the use of suitable dielectric substrates like boron nitride^11^, the decoupling of the substrate by intercalation^12^, or the preparation of suspended graphene^13^. Moreover, the proximity effect can be deliberately exploited to specifically tune the properties of a graphene sheet^14–18^. For example, the almost negligible spin-orbit coupling can be significantly increased by bringing the graphene layer into contact with transition metal dichalcogenides^14,15^ and proximity superconductivity can be observed in graphene in the vicinity of superconducting materials^19^.

In the context of charge transport in epitaxial graphene, a locally varying potential landscape and a spatially inhomogeneous current density are induced by local defects like substrate steps and local variations of the coupling between the graphene layer and the substrate. Analyzing the published results for resistances assigned to specific defects in epitaxial graphene, one finds a large spread^4–7,20–25^. The strong variation in the experimental values of sheet or defect resistances determined by local probe measurements is likely due to the lack of information about the actual local current density. Replacing the probe by a single-electron transistor allows simultaneous measurement of local voltage drop and current distribution in 2D materials^26^ with a lateral resolution in the range of 350 nm^26^. In comparison, STP has an angstrom resolution^27^ and measures the local electrochemical potential with high accuracy, but local variations in the current density are experimentally not accessible and are indistinguishable from spatial variations of the sheet resistance. For conventionally grown graphene on SiC(0001), typically monolayers as well as bilayers are present. Monolayer-bilayer transitions represent strong scattering centers and cause a significant variation of the local current density. Having no better approach, so far local transport properties have been determined using an averaged (sometimes even macroscopic) current density for the whole sheet.

In this study, we show that the high quality of epitaxial monolayer graphene samples grown by polymer assisted sublimation growth (PASG) opens a promising way to quantify also delicate local transport properties with high precision. Applying the PASG method, it is possible to grow large-scale monolayer graphene sheets without bilayer formation^9,28^ on SiC substrates with ultra-low step heights. This allows for local transport investigations of monolayer graphene on terraces with different SiC terminations free from bilayer and step edge effects.

The aim of this work is to test for local variations in the sheet resistance of epitaxial graphene and to unravel possible intrinsic proximity effects induced by the presence of the substrate.

## Results

### Homogeneity of the current density

**Fig. 1:**
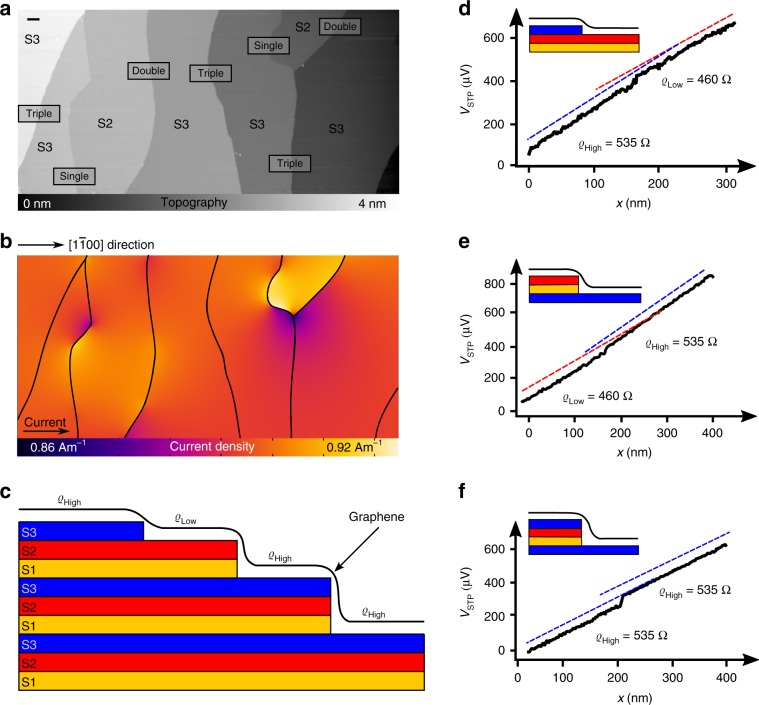
Current density and evaluation of the local sheet resistance at room temperature. **a** Large-scale constant current topography (2 µm × 1 µm, $V_{{\mathrm{Bias}}} = 0.5\;{\mathrm{V}},\;I_{\mathrm{T}} = 0.03\;{\mathrm{nA}}$). S1, S2, and S3 indicate the fundamental bilayers (and thus the stacking) of the SiC substrate, details are given in the discussion. STP measurements were performed in the marked areas (black boxes). The height of the steps is denoted in the marked areas, the scale bar is 100 nm. **b** Using the macroscopic ohmic resistance, the sample geometry shown in **a** and step resistances of 6 Ωμm, 12 Ωμm, 18 Ωμm for single, double, and triple steps, respectively, the $[1\bar 100]$ component of the local current density $j_{{\mathrm{local}}}\left( {x,y} \right)$ is calculated with a finite element simulation. **c** Schematic side view of the crystal structure of 6H-SiC. **d** Measured voltage drop along the graphene layer induced by the cross voltage *V*_cross_ when crossing a single step. Dashed lines represent the slope of the voltage drop (shifted for clarity). **e** Corresponding measurement for a double step and **f** for a triple step.

The local electric field as well as the local current density are needed to determine the local sheet resistance. While STP measures the local voltage drop, the local current density is a priori unknown. In STP studies, it is replaced by an averaged value, e.g., given by the total current and the geometry of the sample. While this approximation has severe limits for locally inhomogeneous samples, the excellent lateral homogeneity of the PASG graphene parallel to the steps, the absence of bilayer graphene and the low impact of steps on the overall resistance^28^, drastically reduce lateral current density variations^29^. In our STP setup, the current flow was deliberately driven parallel to the miscut of the SiC sample, resulting in an overall voltage drop perpendicular to the substrate steps ($[1\bar 100]$ direction). The experimental geometry and the assumption that graphene terraces have a constant sheet resistance parallel to the steps result in a constant average current density on all terraces. To estimate the remaining variation in $j_{{\mathrm{local}}}\left( {x,y} \right)$, we have modeled the local current density for a given surface geometry (Fig. 1a) taken from constant current topographies (CCT) with a resistor network^6,22,30^ and find that the resulting current density exhibits a maximum variation of up to 7% (Fig. 1b). By carefully selecting regions away from complex step configurations, e.g., convergence of two steps, the current density can be considered as highly homogeneous (compare Supplementary Table 1). It is $j_{{\mathrm{local}}}\left( {x,y} \right) \approx j_{{\mathrm{local}}} = \left( {0.89 \pm 0.01} \right)\;{\mathrm{Am}}^{ - 1}$ for an applied voltage along the graphene layer perpendicular to the substrate steps of 1V at *T* = 300K. Comparing the lateral variation of the current density in PASG graphene samples with conventionally grown epitaxial graphene, it becomes obvious that local variations in ${\it{\upvarrho }}_{{\mathrm{sheet}}}$ from monolayer and bilayer graphene and monolayer–bilayer junctions in conventionally grown epitaxial graphene induce a strong variation of $j_{{\mathrm{local}}}(x,y)$ (Supplementary Fig. 1).

### Local variation of $\varrho _{{\mathbf{sheet}}}$ at temperature *T* = 300K

Large scale constant current topographies (Fig. 1a) reveal a surface with single, double as well as triple substrate steps and no bilayer regions as expected for epitaxial graphene grown by PASG^9,10,28^. STP measurements investigating ${\it{\upvarrho }}_{{\mathrm{sheet}}}$ are performed across all step configurations in Fig. 1a, the corresponding voltage drops *V*_STP_ are shown in Fig. 1d–f. Interestingly, to the left and to the right of single substrate steps we find a different gradient of *V*_STP_ (Fig. 1d), indicated by the dashed blue and red lines representing the slope to the left and to the right of the step, respectively. Since the current density is constant, this directly proves that the top and bottom terrace have different sheet resistances. This finding also holds for terraces connected by a double substrate step (Fig. 1e), whereas the identical ${\it{\upvarrho }}_{{\mathrm{sheet}}}$ is measured when crossing a triple substrate step (Fig. 1f). For all step configurations, an additional voltage drop at the topographic position of the step is observed, which is commonly explained by a potential barrier induced by the step due to detachment of the graphene sheet from the substrate^4,5,31^.

In order to further investigate spatial variations of ${\it{\upvarrho }}_{{\mathrm{sheet}}}$, we have measured large sequences of steps. The topographic analysis has shown that instead of a random distribution of step heights, a well-defined sequence of the step heights shows up: along the $[1\bar 100]$ direction, either a triple substrate step is present or a single substrate step and a double substrate step are observed. These characteristic step patterns for PASG graphene on 6H-SiC have recently been reported in an Atomic Force Microscopy (AFM) study and have been attributed to the growth process^28^. The detailed STP analysis of large sequences of substrate steps allows deducing two implications: firstly, the evaluation shows that at 300K the sheet resistance across a given terrace is constant (Supplementary Fig. 2). Secondly, from STP measurements on more than 40 terraces, we extract two clearly distinct sheet resistances, which we refer to as ${\it{\upvarrho }}_{{\mathrm{High}}}$ and ${\it{\upvarrho }}_{{\mathrm{Low}}}$. The mean ${\it{\upvarrho }}_{{\mathrm{High}}}$ is 535Ω and the mean ${\it{\upvarrho }}_{{\mathrm{Low}}}$ is 460Ω. The mean ${\it{\upvarrho }}_{{\mathrm{High}}}$ and ${\it{\upvarrho }}_{{\mathrm{Low}}}$ deviate by $\left( {14 \pm 1} \right)\%$ from each other at room temperature. Moreover, ${\it{\upvarrho }}_{{\mathrm{High}}}$ as well as ${\it{\upvarrho }}_{{\mathrm{Low}}}$ show a variation from terrace to terrace of ± 20Ω.

### Temperature-dependence of $\upvarrho _{{\mathbf{sheet}}}$

**Fig. 2:**
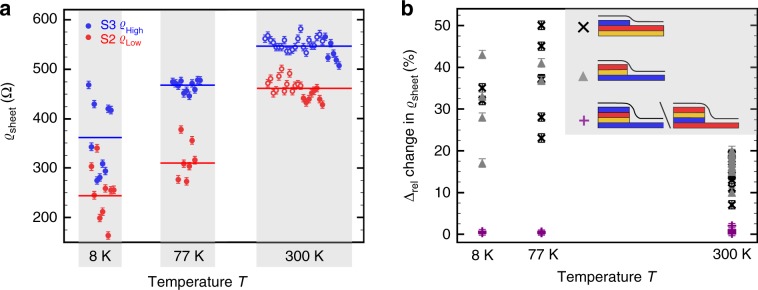
Temperature-dependence of the sheet resistance. **a** Sheet resistance at 8 K, 77 K, and 300 K acquired on more than 80 terraces for two samples (indicated by open and filled symbols), solid horizontal lines indicate the mean value for a given terrace and temperature. **b** Change in sheet resistance for adjacent terraces for all three cases S3→S2, S2→S3, and S3→S3/S2→S2 as a function of temperature. Error bars indicate the experimental measurement uncertainty of the individual data points. Source data are provided as a Source Data file.

In order to disentangle possible scattering processes and to understand the difference between ${\it{\upvarrho }}_{{\mathrm{High}}}$ and ${\it{\upvarrho }}_{{\mathrm{Low}}}$, we performed further temperature-dependent STP measurements at 77K and 8K (Fig. 2a). In this study two different samples, both prepared using the PASG method, from two different batches were analyzed as summarized in Fig. 2a. Both samples show good quantitative and qualitative agreement at room temperature, low-temperature measurements were carried out on one of the two samples. This allows on the one hand to compare results for different samples and on the other hand to discuss the temperature dependence of the sheet resistance for one given sample.

We find an overall decrease in the sheet resistance with decreasing temperature, which is supported by macroscopic transport measurements in four-point van der Pauw geometry (Supplementary Fig. 3) and in agreement with published results^32,33^. The relative reduction in ${\it{\upvarrho }}_{{\mathrm{High}}}$ with decreasing temperature is slightly smaller, i.e., it reduces by 32%, from 535Ω to approximately 365Ω at 8K, compared with the temperature-dependence of ${\it{\upvarrho }}_{{\mathrm{Low}}}$, which declines from 460Ω to an average value of 250Ω at 8K, i.e., it reduces by 45%. Besides the overall reduction of ${\it{\upvarrho }}_{{\mathrm{sheet}}}$, a surprising large increase in the spread in the data is observed with decreasing temperature. At 8K, a maximum variation in ${\it{\upvarrho }}_{{\mathrm{sheet}}}$ of ~270% between the lowest value for ${\it{\upvarrho }}_{{\mathrm{Low}}}$ and the highest value for ${\it{\upvarrho }}_{{\mathrm{High}}}$ is observed. On adjacent terraces a maximum variation of 178% (Supplementary Fig. 4) is measured. In the following, we will use $\Delta _{{\mathrm{rel}}} = ({\it{\upvarrho }}_{{\mathrm{sheet}}1} - {\it{\upvarrho }}_{{\mathrm{sheet}}2})/({\it{\upvarrho }}_{{\mathrm{sheet}}1} + {\it{\upvarrho }}_{{\mathrm{sheet}}2})/2$ to quantify the relative change in the sheet resistance for adjacent terraces (Fig. 2b). Regardless of the temperature, when crossing a triple substrate step, the variation in ${\it{\upvarrho }}_{{\mathrm{sheet}}}$ is small, i.e., $\Delta _{{\mathrm{rel}}} \,<\, 3\%$. In contrast to this, the relative variation in ${\it{\upvarrho }}_{{\mathrm{sheet}}}$ to the left and to the right of single and double substrate steps increases when going from 300K to 77K. In particular, for terraces connected by single or double substrate steps a mean relative change of more than 30% is measured. In both cases Δ_rel_ slightly decreases from 77K to 8K.

### Analysis of the surface morphology of steps and terraces

**Fig. 3:**
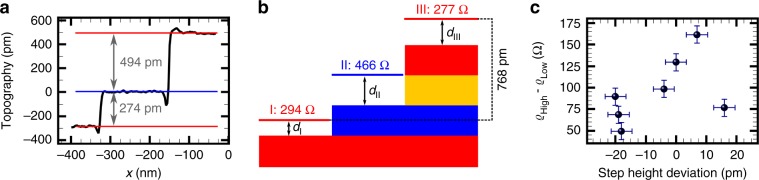
Analysis of the step height of single and double steps. **a** Line profile through a constant current topography showing adjacent terraces S2, S3, S2, connected by a single substrate step followed by a double substrate step recorded at 77K. The line profile reveals a deviation from the step heights of the SiC substrate steps. **b** Schematic representation of the correlation between step height and sheet resistance illustrating a locally varying distance *d* between the graphene layer and the substrate. **c** Difference in the sheet resistance for adjacent terraces for single and double steps measured at 8K as a function of the deviation of the step height. Details are given in the “Discussion”. Error bars indicate the experimental measurement uncertainty of the individual data points. Source data are provided as a Source Data file.

To further investigate the local variation of the transport properties, structural and electronic properties of PASG graphene have been analyzed on different length scales on the same samples. On a mesoscopic scale the surface is characterized by single, double and triple steps, resulting from the miscut of the SiC substrate. Surprisingly, we rarely found the expected height of the substrate steps, i.e., multiple of 0.25 nm^34^. Instead, we observed deviations of the step height with smaller as well as larger values for both single and double steps. As an example, Fig. 3a displays a line profile across a step sequence consisting of a single substrate step and a double substrate step.

For this specific step configuration, the analysis reveals a step height >0.25 nm for the single substrate step and a step height <0.5 nm for the double substrate step, i.e., also the combined step height does not fit to the expected value of three times 0.25 nm. Assuming that different step heights correspond to different distances between the graphene monolayer and the substrate, step sequences (Fig. 3) allow to study the relation between distance and sheet resistance. As usual for graphene on SiC, also for PASG graphene a buffer layer forms between the SiC surface and the graphene sheet^9^. However, since we cannot pin down the vertical position of the height variation, we use the wording ‘distance to the substrate’.

The corresponding STP measurement reveals a higher conductivity on terrace III compared with terrace I (see Fig. 3b), indicating that a larger distance results in a smaller resistance. Details on the dependence of ${\it{\upvarrho }}_{{\mathrm{sheet}}}$ on the step height are summarized in Supplementary Fig. 5, the general trend is that larger distances result in higher conductivities. Moreover, comparing terraces connected by steps with almost identical step height (e.g., Supplementary Fig. 5 black: 507 pm and pink: 500 pm), we find a large spread of the sheet resistances: 304Ω vs. 465Ω and 165Ω vs. 294Ω for the black and pink configuration, respectively (see also Supplementary Fig. 6a–c). Height deviations are found for all temperatures (Fig. 3a, Supplementary Figs. 5 and  6d, e) and the topographic nature of the observed height deviation in CCT is supported by AFM topographies (Supplementary Fig. 7). Details of the height analysis are given in Supplementary Figs. 8 and 9.

**Fig. 4:**
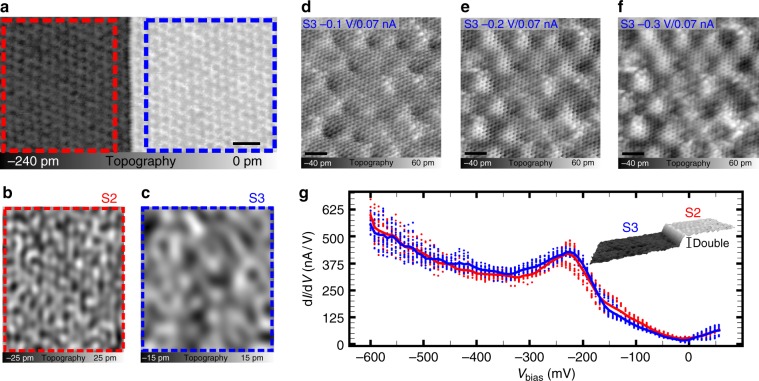
Analysis of the local defect structure on terraces S2 and S3. **a** 50 nm × 25 nm constant current topography of terraces connected by a single substrate step. The scale bar is 5 nm. On both terraces the 6 × 6 modulation is well resolved. The topographic contrast is disentangled into its spectral components (as shown in Supplementary Fig. 11) using Fourier analysis. **b**, **c** The long-range contributions to the constant current topography are shown for the areas in **a** marked with dashed red and blue boxes, respectively. **d** CCT (8 nm × 8 nm, $I_{\mathrm{T}} = 0.07\;{\mathrm{nA}}$) to the left of a double step on a terrace S3 acquired with a bias voltage of $V_{{\mathrm{Bias}}} = - 0.1\;{\mathrm{V}}$, **e** with a bias voltage of $V_{{\mathrm{Bias}}} = - 0.2\;{\mathrm{V}}$ and **f** with a bias voltage of $V_{{\mathrm{Bias}}} = - 0.3\;{\mathrm{V}}$ representing the integral local density of states in the energy interval $E_{\mathrm{F}} \pm eV_{{\mathrm{bias}}}$. For **d**, **e**, **f** the scale bar is 1 nm. **g** Scanning tunneling spectroscopy at 8K on terraces S2 and S3 separated by a double substrate step as indicated in the constant current topography in the inset $\left( {V_{{\mathrm{Bias}}} = - 0.6\;{\mathrm{V}},\;I_{\mathrm{T}} = 0.15\;{\mathrm{nA}}} \right)$. The solid blue line shows the averaging of all spectra recorded on S3 and the solid red line shows the averaging of all spectra recorded on S2.

In order to take the atomic-scale structure of the sample into account, we acquired higher resolved CCTs on terraces connected by single and double substrate steps as shown in Fig. 4a and Supplementary Fig. 10b, respectively. On all terraces the 6 × 6-quasi corrugation (The wording “quasi” modulation is used, because it consists of two types of ring like structures with slightly different size. One large and two smaller rings together form the superstructure^35^.) is visible. It is induced by a lattice mismatch of the graphene sheet and the substrate and originates from actual height corrugation as well as from electronic contrast^35–38^. However, this 6 × 6-quasi corrugation is structurally not perfect (compare Fig. 4a). In order to analyze deviations from a perfect ordering, we disentangle the constant current topographies using Fourier analysis (Supplementary Fig. 11). Applying this type of evaluation for each terrace separately, we disentangle three different contributions to the topographic contrast. Firstly, the 6 × 6-quasi corrugation itself, secondly short-range noise and thirdly, long-range spatial modulations, which can be understood as perturbations of the 6 × 6-quasi corrugation. The latter contributions are shown in Fig. 4b, c for the terraces to the left and to the right of the single substrate step in Fig. 4a, respectively. We determine the dominant wavelength of these modulations as shown in Supplementary Fig. 12 and find a clear difference between the two terraces. The terrace to the left of the single substrate step (Fig. 4b) shows a spatial modulation with a shorter wavelength of 4.2 nm compared with the wavelength of the spatial modulations on the terrace to the right of the single substrate step (Fig. 4c) with 8.1 nm. The corresponding, i.e., reversed finding, holds for terraces connected by a double substrate step (Supplementary Fig. 10b) for which comparable wavelengths of the spatial modulations are extracted. Besides differences in the dominant wavelength, the spatial modulations also exhibit different amplitudes.

In summary, the analysis of the surface morphology allows two conclusions. Firstly, the deviation of the step heights indicates a locally varying distance between the graphene layer and the substrate. Secondly, the 6 × 6-quasi corrugation does not show a perfect ordering.

### Analysis of the local electronic structure

CCTs taken at different bias voltages have additionally been used to gain insight into the local density of states of the combined graphene/buffer layer/SiC substrate system in a given energy interval $E_{\mathrm{F}} \pm eV_{{\mathrm{bias}}}$. Since we cannot separate electronic states originating from the buffer layer from states originating from the SiC substrate, we refer to this part as ‘interface layer’. For epitaxial graphene on SiC it is known that for larger voltages *V*_bias_ electronic states of the interface layer become visible in CCTs^39^. In Fig. 4d–f, we show high-resolution, quasi-simultaneous CCTs recorded at $V_{{\mathrm{bias}}} = - 0.1\;{\mathrm{V}}$, $V_{{\mathrm{bias}}} = - 0.2\;{\mathrm{V}}$ and $V_{{\mathrm{bias}}} = - 0.3\;{\mathrm{V}}$. In all images the graphene honeycomb lattice as well as the 6 × 6-quasi corrugation are well resolved. They dominate the topographic contrast at $V_{{\mathrm{bias}}} = - 0.1\;{\mathrm{V}}$. In contrast, at higher voltages additional states of the interface are visible as non-periodic defect structures, which agrees with published results^39,40^. An example for defects of the SiC substrate is disorder. It has recently been shown by X-ray standing wave analysis^41^ that the top layer of the SiC substrate is Si depleted. This result is in qualitative agreement with a recent HRTEM study^42^ revealing a gradual depletion of Si across the topmost three bilayers. The depletion is due to the partial decomposition of the top SiC layers during the growth process leading to a varying Si concentration. This type of substrate disorder might also be present in PASG graphene.

Spectroscopic measurements using scanning tunneling spectroscopy (STS) at 8K provide insight into the local electronic structure. d*I*/d*V* spectra of graphene on SiC show two prominent minima, firstly the so-called pseudogap at 0 meV and secondly a minimum at the position of the Dirac point^43^. The position of the latter minimum gives a hint to the local charge carrier density^44^. In Fig. 4g the STS data acquired on two terraces connected by a double substrate step are shown. The d*I*/d*V* spectra in Fig. 4g are very similar and in agreement with ARPES measurements (Supplementary Fig. 13) and published results^43,45^. Quantitative deviations between STS and ARPES measurements may be due to different measurement conditions such as the temperature, the addressability of electronic states in the different techniques and due to the presence of the probe itself in STS measurements. In addition to the two prominent minima, we find a pronounced maximum between −200 mV and −250 mV, which we assign to the interface states observed in CCT.

On closer inspection of the individual d*I*/d*V* spectra it can be seen that the electronic properties on the two terraces are not identical and even on a given terrace we find local deviations (Fig. 4g). In order to quantify these deviations, we describe each individual d*I*/d*V* spectrum in the region of the minimum at negative voltages with a polynomial fit (Supplementary Fig. 14). From the minima of these fits we obtain the position of the Dirac point for each spectrum separately. The variations on a given terrace regarding the position of the Dirac point are comparable to the differences in d*I*/d*V* spectra on the two different terraces (Supplementary Fig. 14). For the terrace to the left we find an average value of $E_{\mathrm{D}} = \left( { - 360 \pm 17} \right)\;{\mathrm{meV}}$, for the terrace to the right the mean value is $E_{\mathrm{D}} = \left( { - 355 \pm 13} \right)\;{\mathrm{meV}}$. The error interval is the standard deviation.

## Discussion

In order to interpret the local transport properties of PASG graphene, we correlate the structural and electronic STM/STS information with the local STP measurement and thereby address a number of questions. Firstly, can we assign the two distinct sheet resistances ${\it{\upvarrho }}_{{\mathrm{Low}}}$ and ${\it{\upvarrho }}_{{\mathrm{High}}}$ unambiguously to characteristics of the sample? Secondly, what causes the huge spread in the sheet resistance at low temperature found for both ${\it{\upvarrho }}_{{\mathrm{Low}}}$ and ${\it{\upvarrho }}_{{\mathrm{High}}}$? And finally, can both effects, the differences in ${\it{\upvarrho }}_{{\mathrm{Low}}}$ and ${\it{\upvarrho }}_{{\mathrm{High}}}$ as well as the spread at low temperature, be traced back to the same origin?

In a first step, we assign the specific step structure revealed in large scale topographies (Fig. 1a) to the stacking sequence of the 6H-SiC(0001) substrate. All SiC crystals consist of fundamental layers of silicon and carbon atoms, arranged in tetrahedral coordination^46–48^, referred to as fundamental bilayers. Although the 6H-SiC(0001) exhibits six different (crystal) terminations (labeled as S1, S2, S3 and S1^*^, S2^*^, S3^*^^49^ in Supplementary Fig. 15), only four out of the six possible 6H-SiC terminations are found^28^, because the terraces S1/S1* have a higher decomposition velocity^34,50^ compared with the other terraces and therefore disappear during the growth process. We label the graphene terraces according to the substrate terminations as S2/S2* and S3/S3*. It directly follows, that graphene on terraces S2/S2* exhibits a low sheet resistance and a short-wave spatial modulation of the 6 × 6-quasi corrugation. In contrast, a larger sheet resistance ${\it{\upvarrho }}_{{\mathrm{High}}}$ and long-wave perturbations of the 6 × 6-quasi corrugation are measured on terraces S3/S3*. A systematic difference in ${\it{\upvarrho }}_{{\mathrm{High}}}$ for S3 compared with S3^*^ and in ${\it{\upvarrho }}_{{\mathrm{Low}}}$ for S2 compared with S2^*^ was not observed (Supplementary Fig. 9). Therefore, we refer to S3/S3* as S3 and S2/S2* as S2 in the following (compare Fig. 1c). In summary, we conclude that S2 and S3 are characterized by sheet resistances, which differ by their absolute values as well as by their temperature dependence.

We continue our discussion with a more detailed comparison of the local structural and electronic properties of PASG graphene. In general, a variation in the sheet resistance can be caused by a modified charge carrier density, e.g., in the framework of polarization doping^51,52^, as well as a variation in mobility. STS data allow to estimate a difference in the local doping on adjacent terraces. In order to cause the variation in the sheet resistance of 140% for the given terraces, the change in the doping level is expected to become visible as a significant shift of the position of the Dirac point in the STS data of terrace S2 compared with terrace S3. Since the mean Dirac energy on terrace S2 compared with terrace S3 is only shifted by ≈5 meV (Fig. 4g, Supplementary Fig. 14), we discard a locally varying polarization doping^51,52^ as the main reason for the observed variation of the sheet resistance. Consequently, the local sheet resistance ${\it{\upvarrho }}$ is predominantly governed by the mobility. The latter is the result of a variety of possible scattering mechanisms like e.g., electron–phonon, electron–electron, or electron–defect interaction, which all could be modified by the local structural and electronic properties of the sample.

To disentangle possible scattering processes in PASG graphene, we first take the measured step heights into account assuming that they reflect the distance of the graphene layer to the substrate and correlate them with the local transport properties of S2 or S3 (see Fig. 3b and Supplementary Fig. 6e). Data sets like the one presented in Fig. 3b allow for a comparison of two terraces with the same substrate termination, yet different distances of the graphene to the substrate. They directly show that a larger distance results in a reduced sheet resistance. To further test this hypothesis, we sort the sheet resistances according to the step heights (Supplementary Fig. 5, datasets determined at 8K) and, with a single exception, find a match that larger distances result in a reduced sheet resistance. This finding holds for S2 as well as S3 termination.

While the step height variation is not a priori expected, the observed correlation is not surprising. For epitaxial graphene on SiC(0001), the buffer layer is partially covalently bonded and thus strongly coupled to the SiC substrate^53^, whereas the graphene layer is only weakly coupled^54^ by van der Waals interaction. Nevertheless, the electronic properties of epitaxial graphene are known to be strongly influenced by the substrate. Epitaxial graphene shows a strong n-type doping^1,35,55^ from interface states^51^ and a limited charge carrier mobility^56^ due to substrate-induced scattering^32,33^. Already a decoupling of the substrate by intercalation leads to an increase in mobility^57^, suspended/free-standing graphene shows the highest mobility^13^ and a reduced charge carrier density^58^. We suggest that for a larger distance the graphene layer decouples from the substrate resulting in a reduced impact of the defect states of the interface. Thus, these terraces exhibit an increased mobility and a reduced sheet resistance compared with terraces where the graphene layer is closer, i.e., more strongly coupled to the substrate.

Within the proposed model, we now discuss the temperature-dependence of the sheet resistance, i.e., an increasing conductivity with decreasing temperature. In the semi-classical Boltzmann transport, an increase in the conductivity with decreasing temperature is attributed to freezing out of electron–phonon^59,60^ and electron–electron scattering^61^. In addition, potential scattering, screening, and their interplay have to be considered in the discussion. While all these processes depend on the charge carrier density, the electron–electron scattering has been found to be most dominant at high temperatures and low doping^62^. ARPES measurements reveal a high charge carrier concentration of $n \approx 1 \times 10^{13}{\mathrm{cm}}^{ - 2}$ (Supplementary Fig. 13) and moreover, our STS results imply a mainly homogeneous carrier density. Thus, the impact of electron–electron scattering can be assumed to be constant on all terraces, i.e., it cannot explain the experimentally observed spread of ${\it{\upvarrho }}_{{\mathrm{sheet}}}$.

Since the Fermi wavelength of the electrons roughly corresponds to the wavelength of the potential modulations, we have additionally considered phase-coherent transport phenomena. We predominantly observed classical Lorentz magnetoresistance in macroscopic magnetotransport measurements (Supplementary Fig. 3b), therefore we conclude that weak localization (and phase-coherent transport in general) is only weakly pronounced. Therefore, we do not further consider this effect.

Electron–phonon scattering in graphene on SiC is governed by the contribution from remote interfacial phonons^63,64^. Since the temperature-dependence of the resistance associated with electron–phonon scattering is consistent with our measurements, we attribute a part of the general temperature-dependence to scattering with substrate phonons. Assuming that electron–phonon scattering causes a monotonous decrease of the sheet resistance with decreasing temperature^32,33^, we estimate the phonon contribution ${\it{\upvarrho }}^{{\mathrm{el}} - {\mathrm{phonon}}}\left( T \right)$as the difference between the mean sheet resistance at 300K and the highest measured values at 8K on terraces S3. This estimation yields a phonon contribution of < 100Ω. Besides the general decrease in the sheet resistance, our data show an increase in the spread of the individual measurements at low temperature (Fig. 2a) accompanied by a reduction in the sheet resistance of up to ≈250Ω when going from 300K to 8K. Within our model, the spread in the data primarily originates from the dependence of the sheet resistance on the distance *d* to the substrate. From this, it directly follows that a local modification of the interaction between the graphene sheet and the substrate results in a locally varying mobility. For electron–phonon scattering, one would expect stronger electron–phonon scattering for smaller *d*, which does not agree with the observed behavior (Supplementary Fig. 5). This strongly indicates an additional relevant scattering mechanism besides electron–phonon scattering, explicitly depending on *d*.

Triggered by the observations from CCT, i.e., spatial modulations like the ones observed in Fig. 4a–c, we propose scattering at local defects and potential fluctuations as the additional scattering mechanism: ${\it{\upvarrho }}\left( {T,\;d} \right) = {\it{\upvarrho }}^{{\mathrm{el}} - {\mathrm{phonon}}}\left( T \right) + {\it{\upvarrho }}^{{\mathrm{el}} - {\mathrm{defect}}}(T,d)$. The topographic contrast in highly resolved CCT is dominated by the 6 × 6-quasi corrugation. It is randomly perturbed (Fig. 4) and consequently, each terrace is unique with respect to its defect structure. This deviation from the perfect ordering of the 6 × 6-quasi corrugation induces a random potential scattering. A temperature-dependent impact of potential scattering on the resistivity has been studied for charged impurities^65^. At low temperatures, the impact of Coulomb scattering at charged impurities is reduced due to localization of electrons and associated screening. We propose that the basics of this concept can be transferred to our system, replacing the charged defects by the non-periodic potential fluctuations of the 6 × 6-quasi corrugation (Fig. 4a–c) as well as interface states (Fig. 4d–f). At low temperatures, electrons are trapped in the random potential, thereby screening the spatial modulations of the potential landscape. This results in a weaker potential landscape for the remaining transport electrons, thus contributing to the general reduction in the sheet resistance.

Within the framework of potential scattering it is reasonable that the localization of electrons and associated screening depends on the structural characteristics of the respective terrace, i.e., the exact shape of the random potential landscape. Therefore, the defect structure that is expected to change from terrace to terrace leads to a variation in the potential as well as the screening. This becomes visible as the large spread in the sheet resistance at low temperatures.

Having identified the distance between the graphene and the substrate as an important parameter that controls the sheet resistance in general, the question arises whether this parameter also explains the different sheet resistances of the graphene sheet on terraces S2 compared with terraces S3. Figure 3c shows the difference in the sheet resistance for adjacent terraces S2 and S3 with respect to the experimentally determined step height deviation. It reveals no clear dependence of the variation in the sheet resistance for adjacent terraces S2 and S3 on the height deviation and thus implies that, in addition to the distance dependence of the sheet resistance, intrinsic differences between S2 and S3 exist. Although not providing a comprehensive picture, first indications of these intrinsic differences can be found in the wavelength of the structural modulation (Fig. 4) of S2 and S3, in the local defect structure of the interface layer and by comparing the local *I*(*V*) spectra acquired on S2 and S3 that show slight differences in the spectra at e.g., −300 mV.

In summary, the spatial homogeneity of PASG graphene allows for a quantitative analysis of electronic transport on a local scale. We have shown a direct correlation of the structural as well as the electronic transport properties with the substrate. In particular, PASG graphene shows a locally inhomogeneous sheet resistance, which is governed by both the substrate termination of the SiC and the distance between the graphene layer and the substrate. A locally varying distance to the substrate is accompanied by a variation of the impact of the interface states such that a larger distance leads to a reduced resistance. By analyzing the temperature-dependence of the sheet resistance we have disentangled different scattering mechanisms and have thereby revealed a large inhomogeneity in the sheet resistance at low temperatures associated with the imperfections of the 6 × 6-quasi corrugation and localized defects.

Besides the investigation of fundamental processes in the interaction between substrate and graphene, the reported anisotropy could be exploited in further transport experiments. For example, it is interesting to simply rotate the sample by 90° such that the current is applied parallel to the steps instead of perpendicular. Generally, terraces S2 with ${\it{\upvarrho }}_{{\mathrm{low}}}$ carry more current than terraces S3 with ${\it{\upvarrho }}_{{\mathrm{High}}}$ depending on the ratio of the two sheet resistances (see Supplementary Fig. 16) yielding transport channels with a minimum width of about ten times the Fermi wavelength. Thus, by selecting suitable narrow terraces, quasi 1D electronic transport might be accessible in a 2D sample. In addition, terraces S2 act as nanoscale heat sources and terraces S3 as heat sinks. This enables the investigation of thermal transport in low dimensions. Thus, PASG graphene can be a model system to study the interplay between electronic and heat transport with the aim of improving the performance of thermoelectric devices^66^. In this context, the question arises as to the limitations of the reported effect, i.e., a maximum variation in the sheet resistance of 270% at low temperatures. Such a strong local inhomogeneity of the electronic transport is an important quality in the field of epitaxial graphene. It e.g., implies that nanometer-sized devices could exhibit a local variation in the mobility of up to 270%. We are convinced that the findings of this study can be generalized and should be considered for other 2D materials grown on and in proximity with a substrate.

## Methods

### Sample preparation

Graphene samples investigated in this study were grown on the (0001) Si-terminated face of semi-insulating 6H-SiC wafers with small nominal miscut angle of 0.06° toward $[1\bar 100]$ direction applying the PASG technique^9,28^. The idea of this method is to support the growth process with an external carbon source. A polymer is deposited on the substrate using liquid phase deposition before high-temperature sublimation growth is initialized^9,10,28^. The samples were initially annealed in vacuum ($p \approx 4 \cdot 10^{ - 7}$ mbar, 900 °C, 30 min). The process was proceeded by intermediate annealing in argon ambient (*p* = 900 mbar) at 1200 °C and 1400 °C for 10 and 2 min, respectively. Afterward, the samples were heated directly up to 1750 °C and annealed (6 min) while argon flux was kept at 0 sccm^10^. Samples prepared with this method are almost defect- and bilayer-free and exhibit shallow step heights, as verified in Raman mapping and AFM topographies^9,28^.

### Scanning probe measurements

The experiments were performed in a custom-built low-temperature STM and in a custom-built room temperature STM under UHV conditions (base pressure < 10^−10^ mbar at 300K, 77K, and 8K) using electrochemically etched tungsten tips. STS spectra were acquired using standard lock-in technique and a modulation amplitude of 10 mV. The concept of the STP setup is depicted in Supplementary Fig. 17a. We electrically contact our samples (3 mm × 7 mm) with gold contacts of 50−100 nm thickness by thermal evaporation in a shadow mask procedure in a two-terminal geometry. In order to eliminate surface contamination, the samples are heated up to 400 °C for 30 min after reinsertion into the UHV chamber. A voltage *V*_cross_ is applied across the sample via two gold contacts. The voltage $V_{{\mathrm{STP}}}\left( {x,y} \right)$, which is a measure of the local electrochemical potential, is adjusted such that the net tunnel current *I*_T_ vanishes and is additionally recorded as a function of position. The resulting potential map (Supplementary Fig. 17b) gives access to the voltage drop along the graphene sheet in the investigated sample area. The simultaneously acquired constant current topography (Supplementary Fig. 17c) allows to directly connect transport and topographic information. The local sheet resistance of each terrace is determined from the potential gradient on the terrace and the current density *j* as follows^4^ ${\it{\upvarrho }}_{{\mathrm{sheet}}} = \frac{{dV_{{\mathrm{STP}}}}}{{dx}} \cdot \frac{1}{j} = \frac{{E_{\mathrm{x}}}}{j}$.

### Finite element simulation with COMSOL

The local current density $j_{{\mathrm{local}}}\left( {x,y} \right)$ was calculated using a finite element simulation based on COMSOL multiphysics^®^ using the AC/DC module. As input parameters, we enter the macroscopic ohmic resistance and the global geometry of the sample. Additional topographic information like substrate steps, bilayer regions, and corresponding monolayer-bilayer transitions are included according to the structural information from constant current topographies. Step resistivities used in this study are set to 6 Ωμm, 12 Ωμm, 18 Ωμm for single, double, and triple substrate steps, respectively.

## Supplementary information

Supplementary Information

## Data Availability

The authors declare that all data supporting the findings of this study are available within the paper and its supplementary information files. Source data for Figs. 2a, b and 3c are provided as Source Data file.

## Source data

Source Data
