# Supplementary material for: Substrate induced nanoscale resistance variation in epitaxial graphene
Source: Nat Commun. 2020 Jan 28;11:555. doi: 10.1038/s41467-019-14192-0 (PMC6987157; doi:10.1038/s41467-019-14192-0)
Supplement: Supplementary file 1 — Supplementary Information [file 41467_2019_14192_MOESM1_ESM.pdf]

Supplementary Information

Substrate induced nanoscale resistance variation in epitaxial graphene

Sinterhauf et al.

Supplementary Figures

Supplementary Figure 1

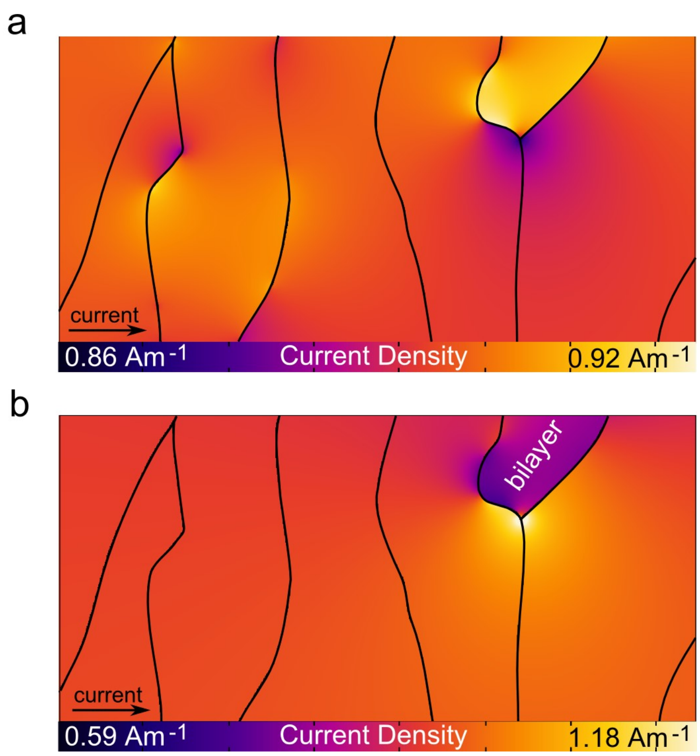

Influence of bilayer regions on the homogeneity of the current density. **a** Using the macroscopic ohmic resistance, the sample geometry and step resistivities of $6\ \Omega\mu\mathrm{m}, 12\ \Omega\mu\mathrm{m}, 18\ \Omega\mu\mathrm{m}$ for single, double and triple steps, respectively, as input parameters, the local current density $j_{\mathrm{local}}(x,y)$ is calculated with finite element simulations using COMSOL for a perfect monolayer grown by PASG. **b** for comparison, a bilayer region with corresponding monolayer-bilayer transition [1] is included in the sample geometry used in a resulting in a highly inhomogeneous local current density. The local variation in the current density increases by almost a factor of ten compared to the pure monolayer case in a as can be seen from the color bars.

Supplementary Figure 2

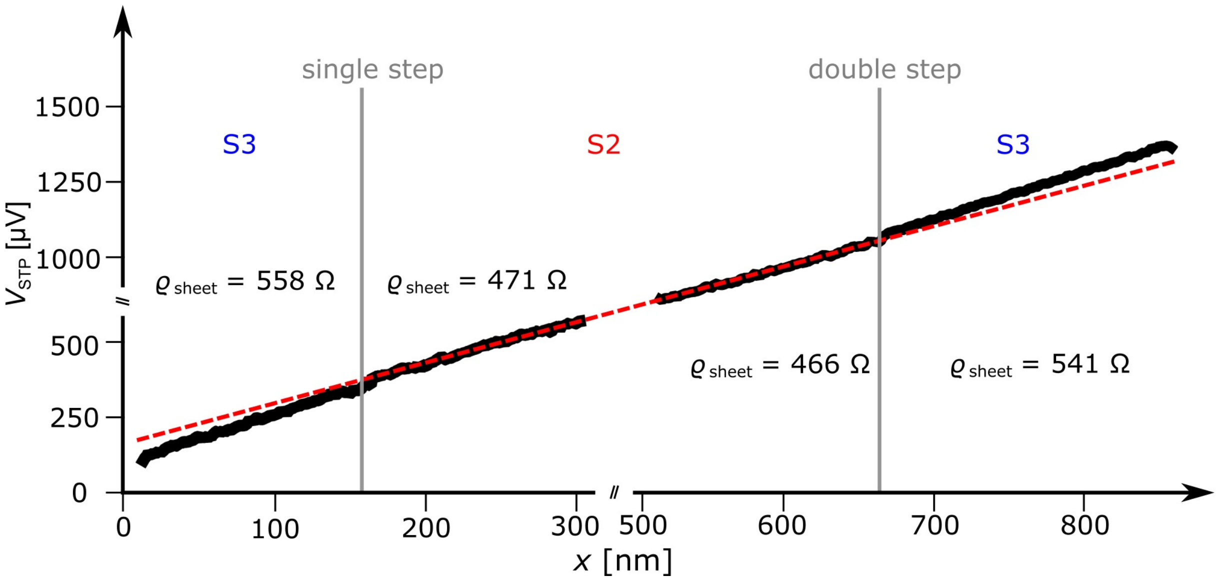

Constant sheet resistance across a given terrace. Voltage drop along the graphene layer for adjacent terraces S3, S2, S3 connected by a single step followed by a double step (left to right). The dashed line represents the slope of the potential in the center region, the variation in the slope in this region is $< 2\%$. Regardless of the measurement position, the sheet resistance can be regarded as constant on a given terrace.

Supplementary Figure 3

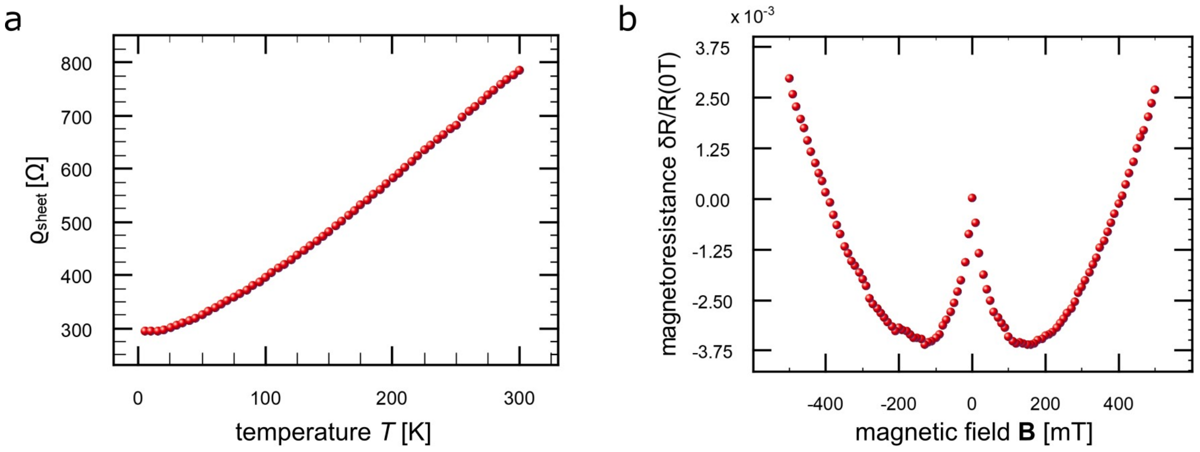

Macroscopic transport measurements in van der Pauw geometry. **a** sheet resistance as a function of temperature in the range of $4\,\mathrm{K}$ to $300\,\mathrm{K}$. **b** magnetoresistance for magnetic fields of $-500\,\mathrm{mT}$ to $+500\,\mathrm{mT}$ acquired at $4\,\mathrm{K}$. At small magnetic fields of up to $\pm 100\,\mathrm{mT}$ a negative magnetoresistance is measured, as also observed for conventionally grown epitaxial graphene [2], which then changes to a classical Lorentz magnetoresistance at larger magnetic fields. We attribute the presence of a negative magnetoresistance at small magnetic fields to weak localization. However, the effect of weak localization is significantly less pronounced than in conventionally grown epitaxial graphene [2]. From this we conclude that phase coherent transport phenomena only play a minor role in the samples investigated in this study.

Supplementary Figure 4

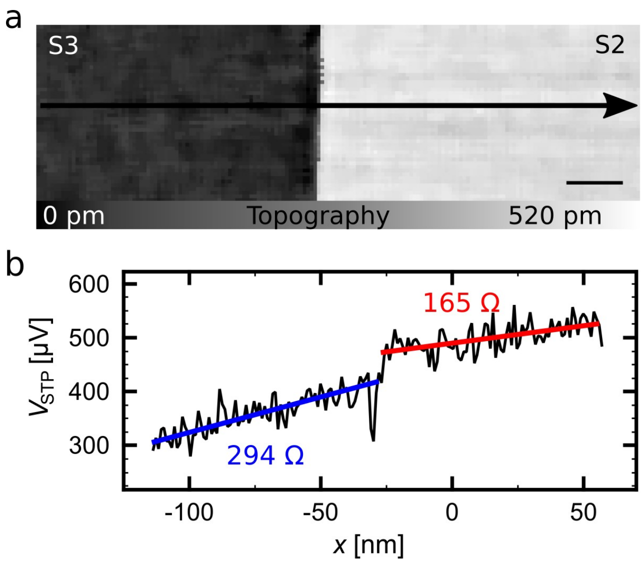

STP data set with a variation in the sheet resistance of 178%. **a** constant current topography (imaging conditions: $V_{\mathrm{Bias}} = 0.03\ \mathrm{V}, I_{\mathrm{T}} = 0.2\ \mathrm{nA}, j = 4.07\ \mathrm{Am}^{-1}$) of monolayer graphene crossing a double substrate step. The scale bar is 15 nm. **b** averaged potential along the black arrow, solid red and blue lines indicate the slope of the potential from which the sheet resistance is calculated.

Supplementary Figure 5

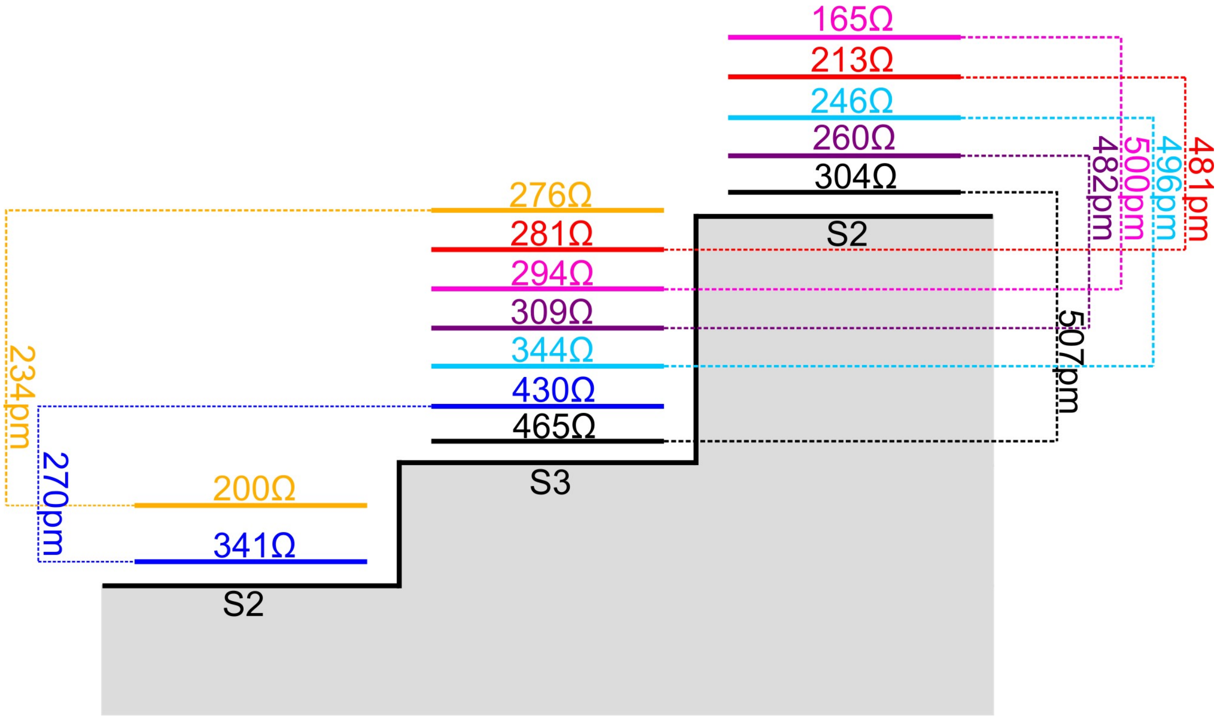

Sorting of the data acquired at $8\ \mathrm{K}$ under the assumption that a larger distance to the substrate leads to a reduction of the resistance. For each terrace, the measured sheet resistances are arranged such that for larger values the distance to the substrate decreases (not to scale). The dotted lines connect adjacent terraces and indicate the

measured step height. By comparing different data sets, predictions for the step height can be made. The pink data set exhibits a step height of $500\ \mathrm{pm}$. Compared to the pink data set, the red data set shows a lower sheet resistance on terrace S3 and a higher sheet resistance on terrace S2. Thus, according to the proposed model, a step height $< 500\ \mathrm{pm}$ is expected for the red data set, which agrees with the measured step height of $481\ \mathrm{pm}$. The only exception is the yellow data set.

Supplementary Figure 6

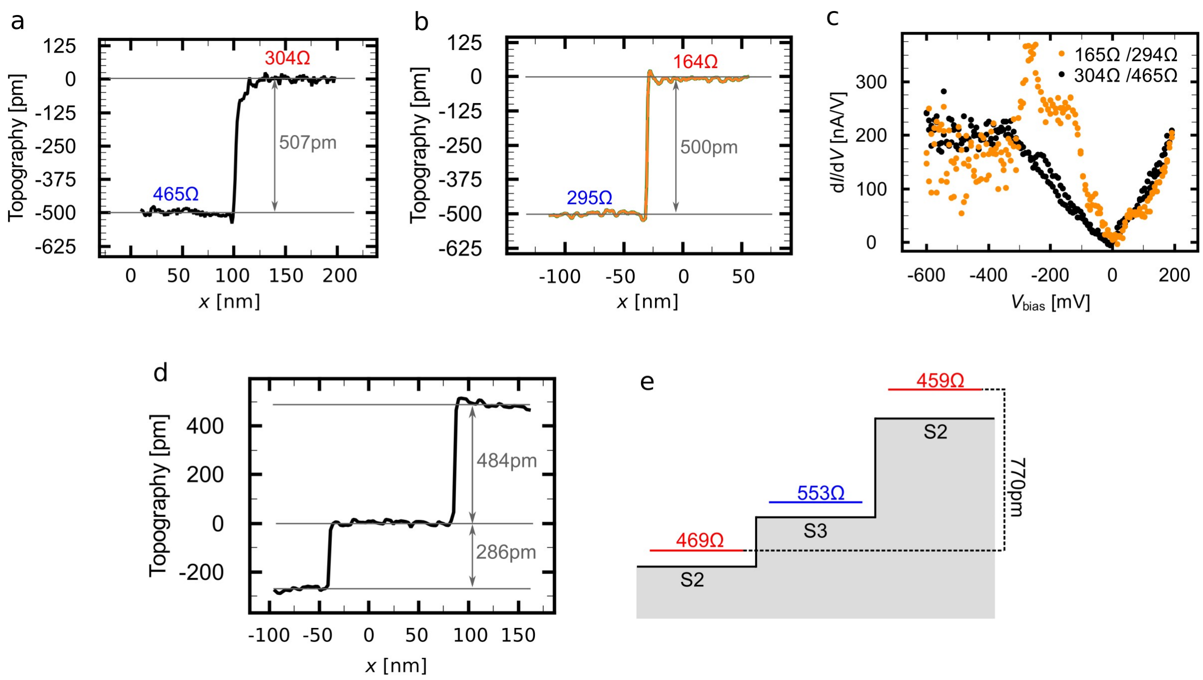

Sheet resistance and step height. **a** sheet resistance and step height for the largest sheet resistance measured at $8\ \mathrm{K}$ **b** sheet resistance and step height for the smallest sheet resistance measured at $8\ \mathrm{K}$. **c** Scanning tunneling spectroscopy corresponding to the data sets shown in a and b. **d** Line profile through a constant current topography ($V_{\mathrm{Bias}} = -0.03\ \mathrm{V}, I_{\mathrm{T}} = 0.2\ \mathrm{nA}$) showing adjacent terraces S2, S3, S2, connected by a single substrate step followed by a double substrate step recorded at $300\ \mathrm{K}$. The line profile reveals a deviation from the step heights of the SiC substrate steps. **e** schematic representation of the correlation between step height and sheet resistance illustrating a locally varying distance between the graphene layer and the substrate.

Supplementary Figure 7

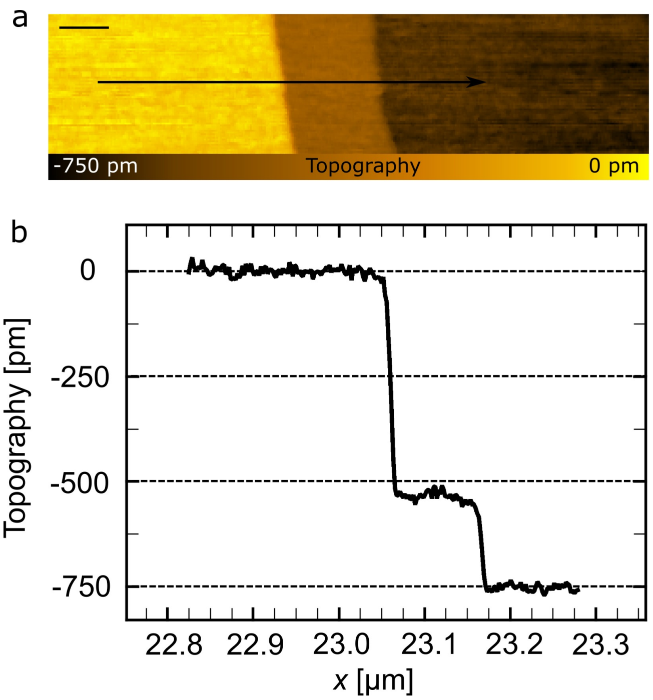

Topographic analysis using atomic force microscopy. **a** AFM topography and **b** line profile along the black line in a reveal a step height < 0.25 nm for the single substrate step and a step height > 0.5 nm for the double substrate step. The scale bar is 50 nm.

Supplementary Figure 8

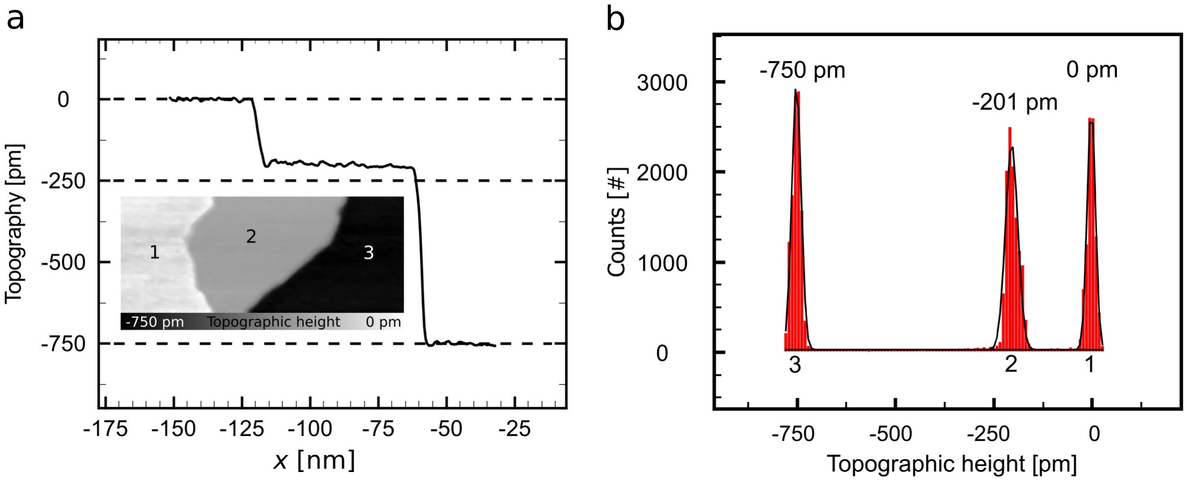

Step height analysis using a histogram method. **a** Line profile through a constant current topography (inset: 600 nm x 100 nm, $V_{\mathrm{Bias}} = -0.03\ \mathrm{V}, I_{\mathrm{T}} = 0.2\ \mathrm{nA}$, the scale bar is 5 nm) showing adjacent terraces S3, S2, S3, connected by a single substrate step followed by a double substrate step. **b** height analysis based on evaluating the height information of each pixel. Gaussian curves are fitted to the peaks, the center position of the individual fits are denoted.

Supplementary Figure 9

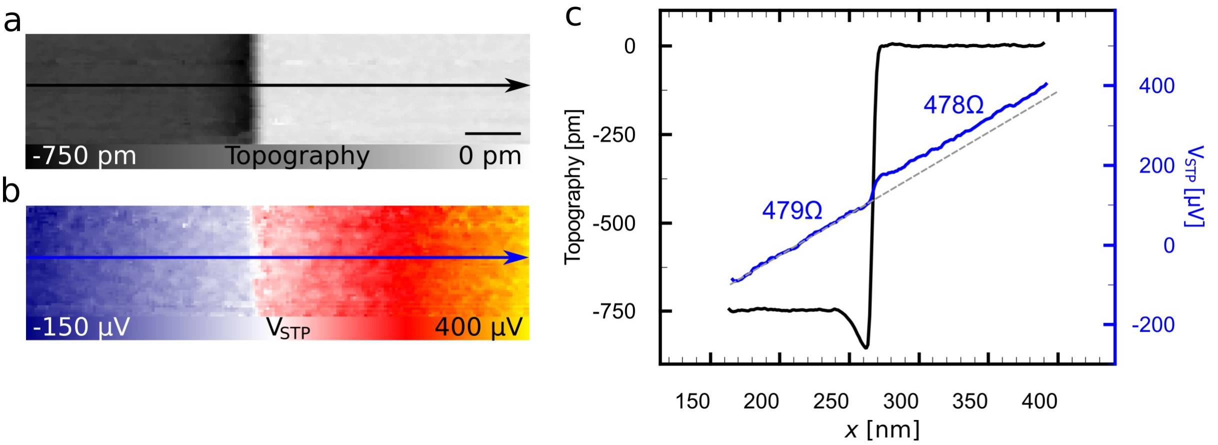

Height calibration using a triple substrate step with almost identical sheet resistance to the left and to the right. **a** constant current topography (imaging conditions: $V_{\mathrm{Bias}} = 0.03\,V, I_{\mathrm{T}} = 0.2\,nA, j = 4.08\,\mathrm{Am}^{-1}$) of monolayer graphene crossing a triple substrate step, **b** simultaneously recorded potential map. The scale bar is 25 nm. **c** topographic height averaged along the black arrow in a and averaged potential along the blue arrow in b. The fact that triple steps show a step height of $750\ \mathrm{pm}$ confirms the correct height calibration of the piezo. The calibration was checked for all examined temperatures at several triple substrate steps.

Supplementary Figure 10

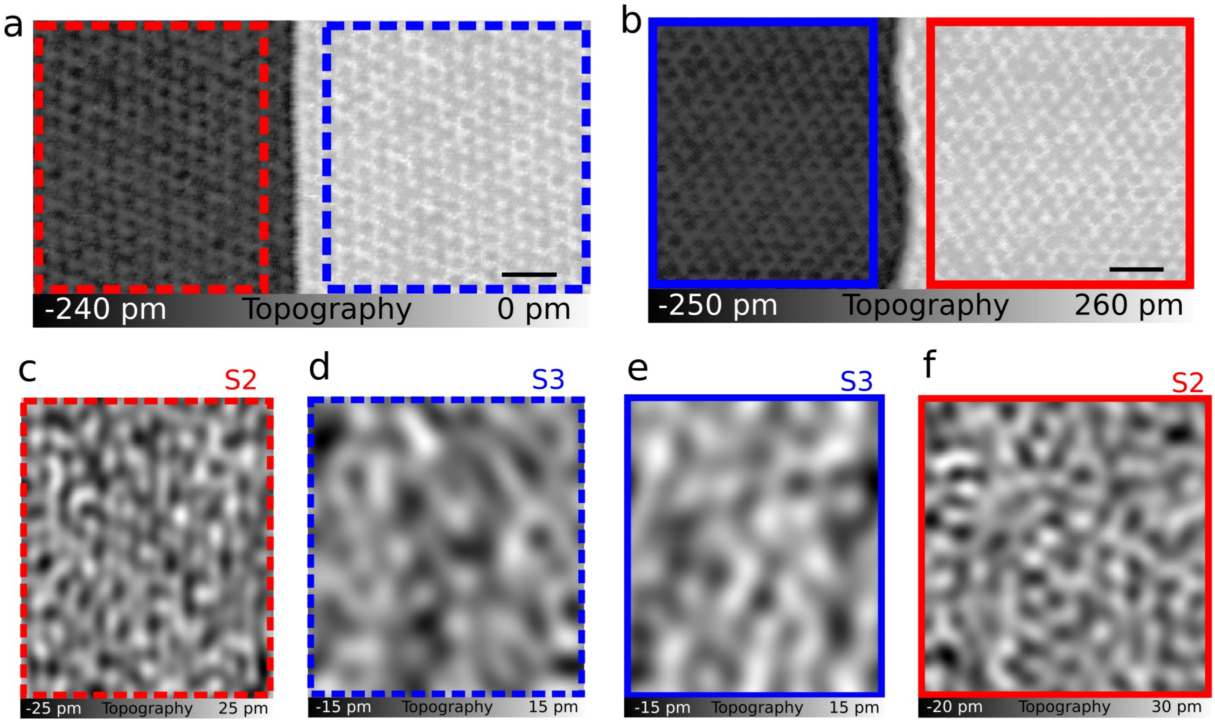

Analysis of the topographic contrast on terraces S2 and S3. **a** 50 nm x 25 nm constant current topography of terraces connected by a single substrate step and **b** connected by a double step ($V_{\mathrm{Bias}} = -0.3\ \mathrm{V}, I_{\mathrm{T}} = 0.15\ \mathrm{nA}$). On all four terraces the 6×6 modulation is well resolved. The topographic contrast is disentangled into its spectral

components (as shown in Supplementary Fig. 11) using Fourier analysis. The scale bar in a and b is 5 nm. In **c** and **d** only the long-range contributions to the constant current topography are shown for the areas in b marked with dashed red and blue boxes, respectively. **e** and **f** depict the corresponding long-range contributions for the areas in c marked with solid red and blue boxes.

Supplementary Figure 11

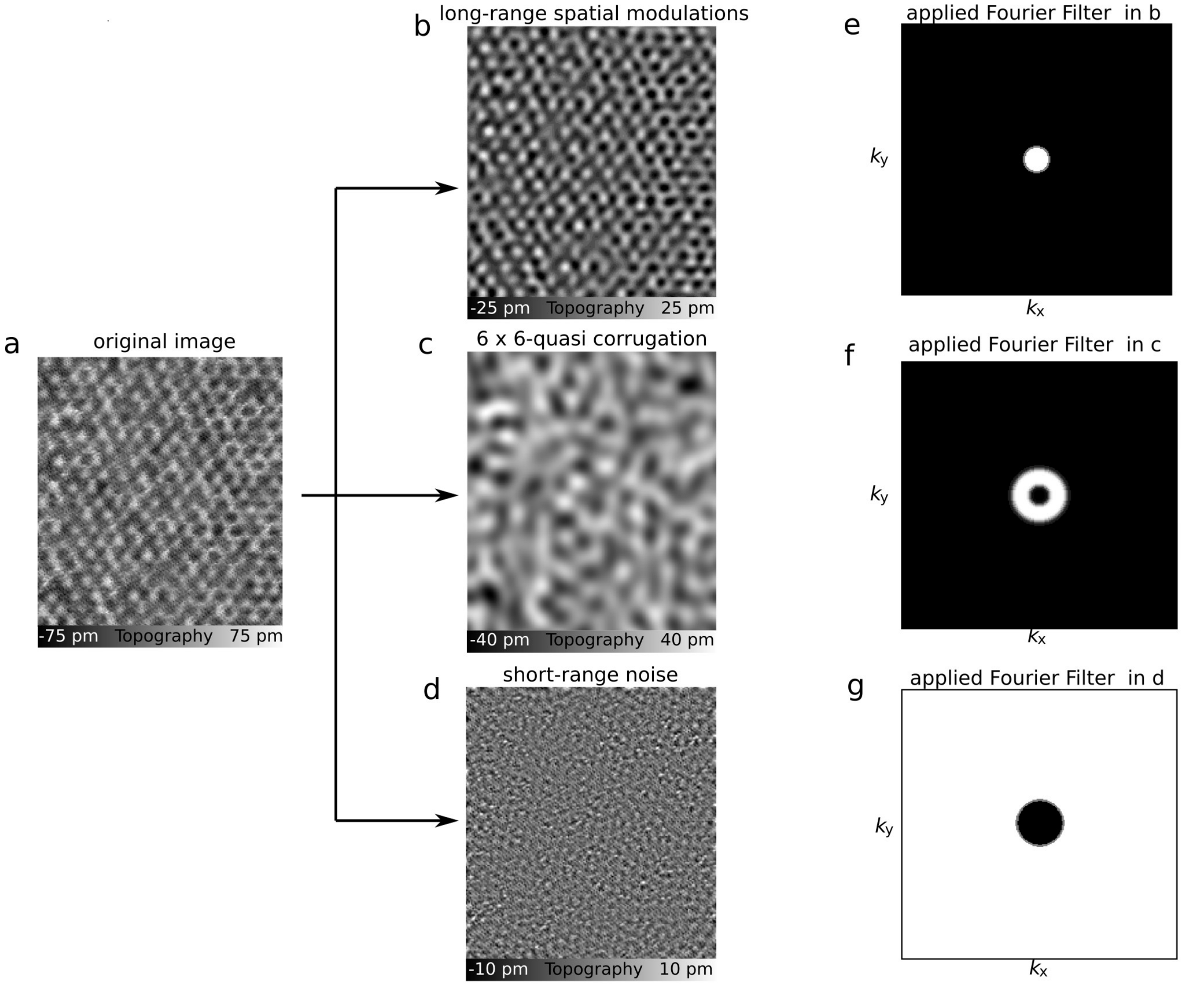

Spectral disentanglement of constant current topographies. **a** original image is disentangled into its spectral components using different Fourier filters: **b** long-range spatial modulation, **c** the 6×6-quasi corrugation, **d** and short-range noise. **e, f** and **g** applied Fourier filters in b, c and d, respectively. Dark regions indicate spectral components that are filtered out.

Supplementary Figure 12

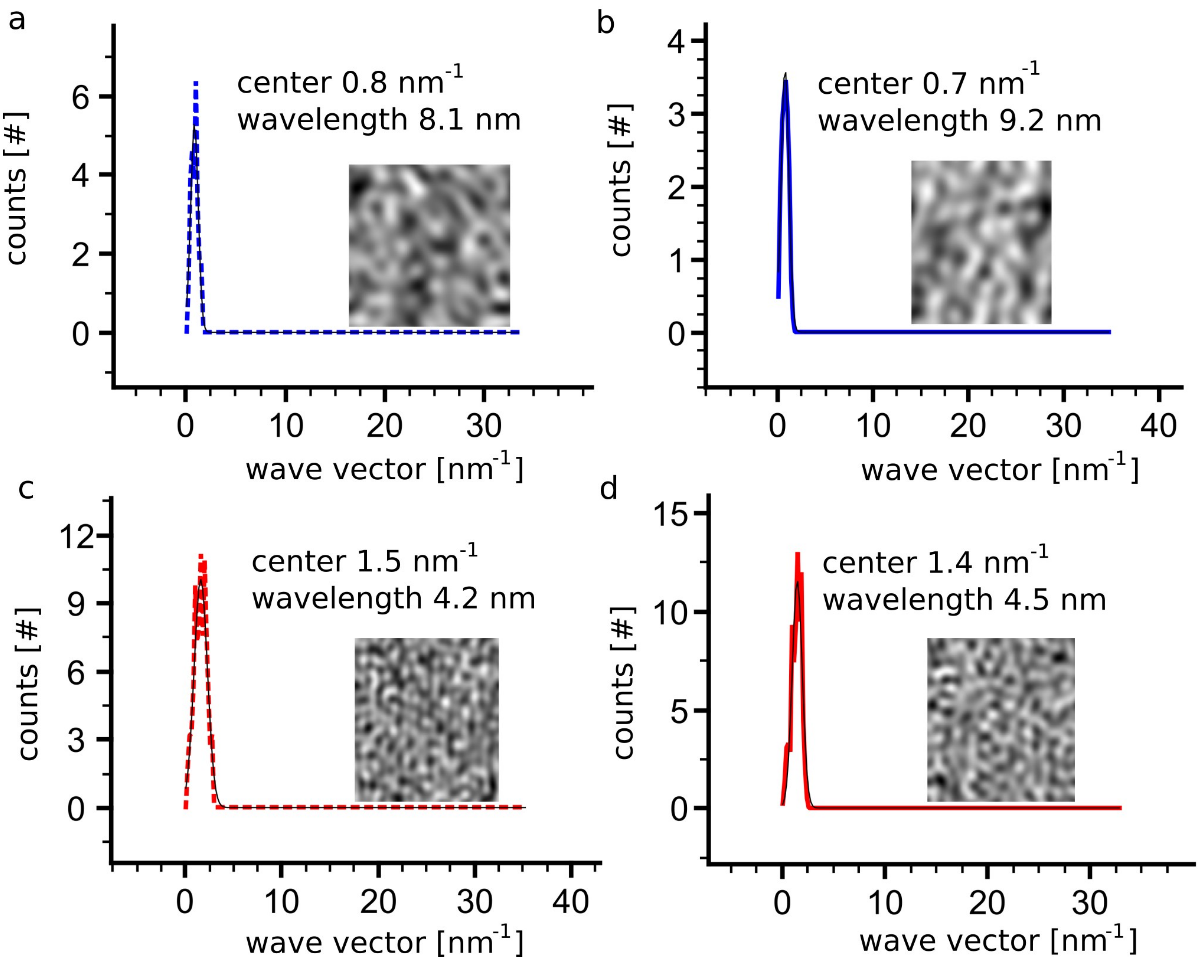

Spectral analysis of the long-range spatial modulations. **a** spectral analysis of the upper terrace in Fig. 4a, **b** spectral analysis of the lower terrace in Fig. 4a. **c,d**, spectral analysis of the topographic contrast in Supplementary Fig. 10b. The original CCTs are Fourier filtered as shown in Supplementary Fig. 11. The resulting long-range contributions are converted into powerspectra for each terrace separately. The wavelength of the spatial modulation is calculated from the center position of a Gauss-Fit adjusted to each powerspectrum.

Supplementary Figure 13

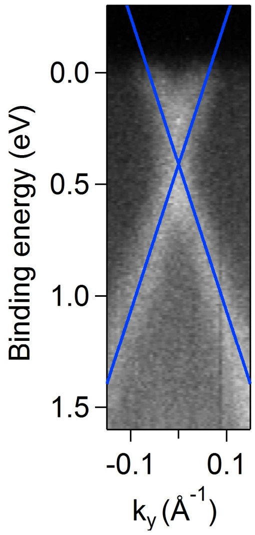

ARPES measurements of the π-bands near E_{F} at the K-point of the graphene Brillouin zone. The photon energy was $\hbar\omega = 40.81\ \mathrm{eV}$. The blue lines correspond to fitted tight-binding bands and the resulting Dirac energy is $E_{\mathrm{D}} - E_{\mathrm{F}} = 410\ \mathrm{meV}$.

Supplementary Figure 14

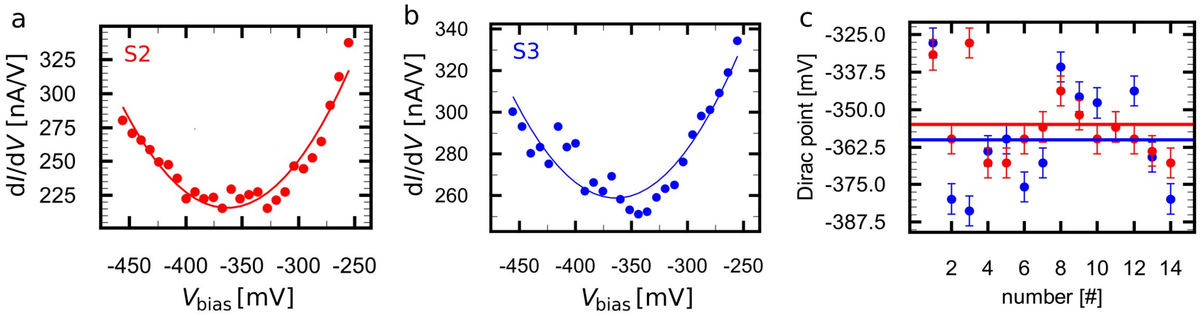

Evaluation of the position of the Dirac point. **a** close-up of a single spectrum recorded on a terrace S2 in the voltage range of $-250\ \mathrm{mV}$ to $-450\ \mathrm{mV}$. The solid line shows a polynomial fit. The position of the Dirac point is given by the position of the minimum of the polynomial fit. **b** close-up of a single spectrum acquired on a terrace S3 and corresponding fit. **c** determined Dirac points for all dI/dV spectra shown in Fig. 4g. On terraces S2 we find an average value of $E_{\mathrm{D}}^{\mathrm{S2}} = (-355 \pm 13)\ \mathrm{meV}$, on terraces S3 the mean value is $E_{\mathrm{D}}^{\mathrm{S3}} = (-360 \pm 17)\ \mathrm{meV}$ as indicated by the solid lines. The denoted error interval is the standard deviation.

Supplementary Figure 15

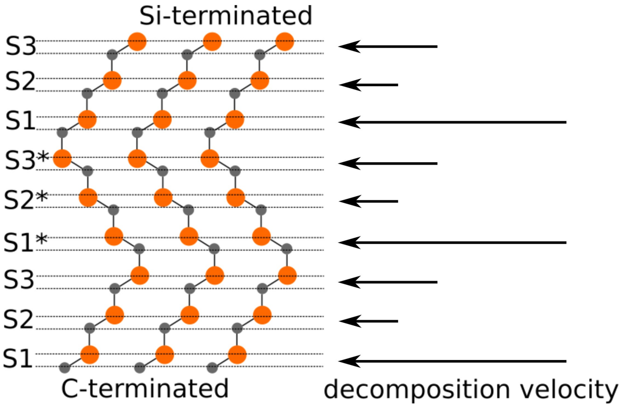

Crystal structure of 6H-SiC(0001). Schematic side view of the crystal structure of 6H-SiC and the decomposition velocity according to [3]. For the decomposition velocities, there are different conclusions in literature as to whether terraces S2/S2* or terraces S3/S3* show a higher decomposition velocity, compare [3] and [4]. However, there is agreement that S1/S1* are the terraces with the highest decomposition velocity and thus disappear first during the growth process.

Supplementary Figure 16

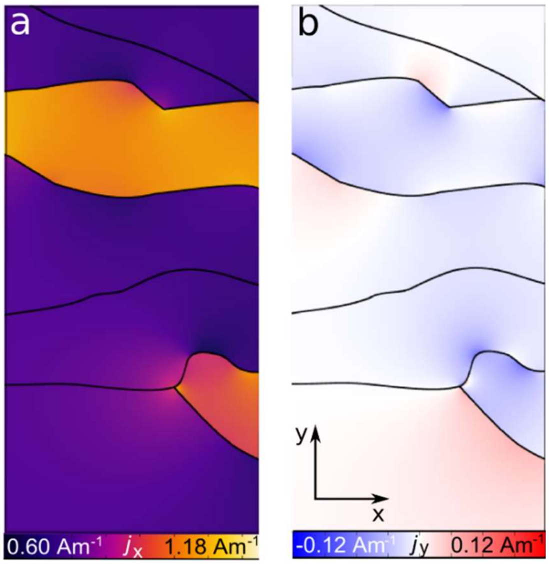

Intrinsic quasi 1D current channels. **a** x-component of the current density from finite element simulation with current flow parallel to the substrate steps. In addition to the sample geometry and the step resistances, each terrace has been assigned a sheet resistance ϱ_{sheet} according to the underlying SiC crystal surfaces. **b** y component of the current density.

Supplementary Figure 17

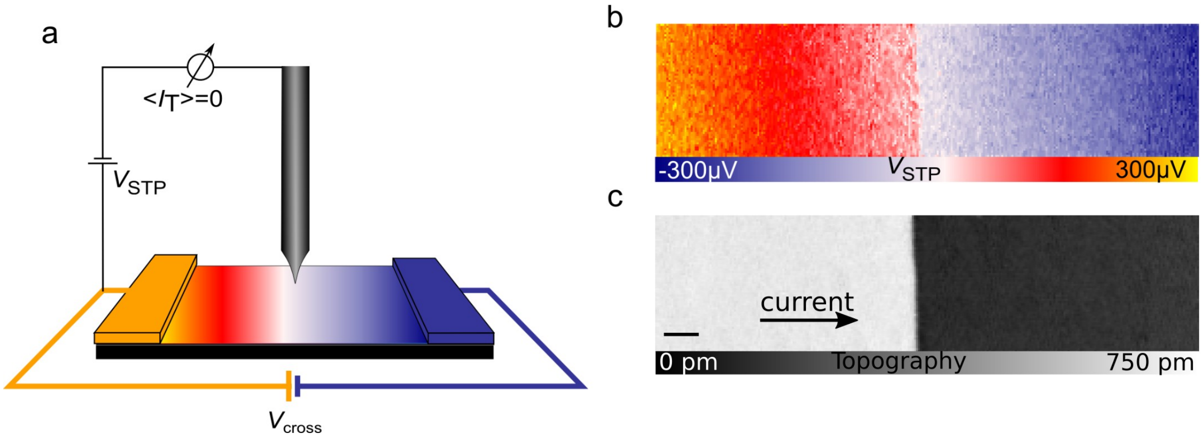

Working principle of our STP setup. **a** schematic drawing of the STP setup: a graphene sample is contacted in two-terminal geometry and a voltage V_{cross} is applied across the sample. The voltage $V_{\mathrm{STP}}(x, y)$ is adjusted such that the net tunnel current I_{T} vanishes. It is recorded at every position of the topography and represents the electrochemical potential of the sample at the position of the tip. **b** resulting potential map and **c** simultaneously recorded $(200 \times 50)\,\mathrm{nm}^2$ topography (imaging conditions: $V_{\mathrm{Bias}} = 0.03\,\mathrm{V}, I_{\mathrm{T}} = 0.15\,\mathrm{nA}, j = 3.56\,\mathrm{Am}^{-1}$) of monolayer graphene crossing a triple substrate step. The scale bar is 10 nm.

Supplementary Tables

measurement	1	2	3	4	5	6	7
current density $[\mathbf{Am}^{-1}]$	0.880	0.885	0.885	0.880	0.882	0.882	0.891

Supplementary Table 1 | Evaluation of the current density. Current densities for all marked areas in Fig. 1a (from left to right) determined from finite element simulations. The macroscopic average current density is $j = 0.89\,\mathrm{Am}^{-1}$ per applied volt cross voltage V_{cross}.

Supplementary References

[1] Willke, P., Druga, T., Ulbrich, R. G., Schneider, M. A., Wenderoth, M., Spatial extend of Landauer residual-resistivity dipole in graphene quantified by scanning tunnelling potentiometry, *Nature Commun.* **6**, 6399 (2015)

[2] Willke, P. et al., Doping of Graphene by Low-Energy Ion Beam Implantation: Structural, Electronic, and Transport Properties, *Nano Lett.* **15**(8), 5110-5115 (2015)

[3] Yazdi, G. R. et al., Growth of large area monolayer graphene on 3C-SiC and comparison with other SiC polytypes, *Carbon* **57**, 477-484 (2013)

[4] Borovikov, V., Zangwill, A., Step bunching of vicinal 6H-SiC{0001} surfaces, *Phys. Rev. B* **79**, 245413 (2009)
